# Risk of Higher Blood Pressure in 3 to 6 Years Old Singleton Born From OHSS Patients Undergone With Fresh IVF/ICSI

**DOI:** 10.3389/fendo.2022.817555

**Published:** 2022-07-05

**Authors:** Yimin Zhu, Yanling Fu, Minyue Tang, Huanmiao Yan, Fanghong Zhang, Xiaoling Hu, Guofang Feng, Yu Sun, Lanfeng Xing

**Affiliations:** Department of Reproductive Endocrinology, Women's Hospital, School of Medicine, Zhejiang University, Hangzhou, China

**Keywords:** OHSS, art, child development, blood pressure, IVF (ICSI)

## Abstract

**Background:**

A large registry-based study found the increasing disorders of cardiovascular and metabolism in IVF children but underlying mechanism is still unknown. Few studies have investigated any association between OHSS and cardiovascular or metabolic function in subsequent children.

**Objective:**

To evaluate the effect of ovarian hyperstimulation syndrome (OHSS) on blood pressure of singletons after *in vitro* fertilization (IVF) with or without intracytoplasmic sperm injection (ICSI).

**Study Design:**

The singlet-center corhort study included 1780 singletons born with IVF/ICSI and 83 spontaneously conceived children from 2003 to 2014. Follow-up has lasted more than 10 years, and is still ongoing. This study analyzed data from follow-up surveys at 3 to 6 years of age.

**Participants, Setting and Methods:**

We recruited 83 children (Group E) spontaneously conceived (SC) as control group and 1780 children born with IVF/ICSI including 126 children born to OHSS-fresh embryo transfer (ET) women (Group A), 1069 children born to non OHSS-ET women (Group B), 98 children conceived by women who developed into moderate or severe OHSS after oocyte retrieval and selected the frozen-thawed embryo transfer (FET) (Group C), 487 children conceived with non OHSS-FET (Group D). We evaluated cardiometabolic function, assessed BP in mmHg, heart rate, anthropometrics, and metabolic index including glucose, serum lipid (triglyceride, total cholesterol, low density lipoprotein, high density lipoprotein), thyroid function, of those children. The BP and heart rate were measured twice on the same day. We applied several multiple regression analyses to investigate the effect of OHSS in the early pregnancy.

**Main Findings:**

By the single factor analysis, the SBP and DBP in the SC group (SBP: 99.84 ± 8.9; DBP: 55.27 ± 8.8) were significantly lower than OHSS-ET group’s, while the blood pressure was similar between the SC group and other three ART groups. Children had higher BP in the OHSS-ET group (SBP: 101.93 ± 8.17; DBP: 58.75 ± 8.48) than in the non OHSS-ET (SBP: 99.49 ± 8.91; DBP: 56.55 ± 8.02) or OHSS-FET group (SBP: 99.38 ± 8.17; DBP: 55.72 ± 7.94). After using multiple regression analysis to adjust current, early life, parental and ART characteristics, the differences in the SBP and DBP (B (95% confidence interval)) between OHSS-ET and non OHSS-ET remained significant (SBP: 3.193 (0.549 to 2.301); DBP: 3.440 (0.611 to 2.333)). And the BP showed no significant difference complementarily when compared non OHSS-FET group with non OHSS-ET group. In addition, the anthropometrics, fast glucose, serum lipid, and thyroid index did not differ among the ART groups.

**Principal Conclusions:**

OHSS might play an independent key role on offspring’s BP even cardiovascular function. Electing frozen-thawed embryo transfer for high risk of OHSS population may reduce the risk of the high BP trend.

**Wider Implications of the Findings:**

It is a large sample study to investigate the effect of OHSS on offspring’s health. These findings provide a clinic evidence of the impact of early environment (embryo even oocyte stage) on the offspring’s cardiovascular health. Our study emphasis the importance of the accuracy of IVF clinic strategy and preventing the OHSS after fresh embryo transfer.

## Introduction

Worldwide, assisted reproductive techniques (ARTs) are used increasingly for those infertile couples. In western industrialized countries, nowadays 1–6% of all newborn children are conceived with the help of IVF with or without ICSI (1). The long-term effect on the development and health of the IVF offspring has aroused people’s attention.

Nowadays a large number of studies in the reproductive area focus on adult chronic diseases which are considered of the fetal origin in the ART offspring, such as cardiovascular diseases, overfat, diabetes, chronic kidney diseases and so on. The cardiovascular disease has still been the most frequently occurring disease, also the leading cause of death in the world. Poorer cardiometabolic outcome after IVF may be due to the higher rates of preterm birth and low birth weight (2), which are risk factors for hypertension and adiposity (3). However, previous studies indicated that poorer cardiometabolic outcome in IVF offspring could not be explained by these factors alone (4–9). As the proposal of the Baker theory (10), there is a shred of increasing study evidence suggesting that the early environment shapes an individual’s health in later life. IVF might compromise the environment of the early embryo (11) as the exposure of high estradiol and progesterone level in the uterus.

As a serious and potentially life-threatening complication of IVF, ovarian hyperstimulation syndrome (OHSS) is characterized by elevated serum estradiol level, massive cystic ovarian enlargement and fluid shift from the intravascular compartment into the third space of the body (12), which has a globally incidence of 23.3% and 1.5% in different published reports (13). In cycles with fresh embryo transplantation, there is still a risk for the development of severe OHSS, which occurs in around 1–14% of IVF cycles (12, 14). OHSS, which shows high estrogen and progesterone during early pregnancy (15, 16) is considered as a good model to study the effects of ovarian stimulation and high sex hormone on the health and development of offspring. Till now far fewer studies have investigated the health and development of the offspring such as cardiometabolic characteristics. Of these studies, some indications of increased cardiovascular and cognitive dysfunction among OHSS-children need further investigation (17, 18).

The objective of this prospective study was to assess the impact of the OHSS in the early pregnancy on BP (Blood Pressure), anthropometrics and some metabolic functions of 3 to 6-year-old singletons. Our primary outcomes were systolic BP (SBP) and diastolic BP (DBP) in mmHg. Secondary outcomes were the measurements of heart rate, weight, standing height, BMI, fasting glucose, parameters of serum lipid metabolism (including total cholesterol (TG), triglyceride (TC), low density lipoprotein (LDL), high density lipoprotein (HDL)) and thyroid function.

## Materials and Methods

### Study Design and Recruitments

For the ART population, this study is a corhort, longitudinal follow-up study of children born to subfertile couples. All the reproductive information of patients was extracted from the patient database of the reproductive center, Women’s Hospital, School of Medicine, Zhejiang University. Between 2003 and 2014, subfertile women who had a successful pregnancy after undergoing IVF/ICSI at the reproductive center of the Women’s Hospital, School of Medicine, Zhejiang University were included in the study. And we randomly recruited SC children born in the above hospital at the same time. **Exclusion criteria**: 1) ART characteristics: sperm or oocyte donation, preimplantation genetic diagnosis/screening (PGD/PGS); 2) couple’s characteristics: woman with hypertension or diabetes before pregnancy, male with a history of hypertension or diabetes, one member of the couple smoked or abused alcohol before pregnancy, one member of the couple had family history of hypertension or diabetes; 3) pregnancy and neonatal outcomes: multiple pregnancy, abortion, fetal loss, stillbirth, neonatal death or with serious diseases; 4) data missing in database. According to Golan and Wasserman’s 2009 criteria (19), besides the abdominal distension, nausea and ovary enlarged, the criteria of moderate or severe OHSS should match at least one of the two following entries in our study: 1) ultrasonic or clinical evidence of ascites or hydrothorax or breathing difficulties; 2) blood volume changed, haemoconcentration, coagulation abnormalities, and renal dysfunction. Then women who satisfied with the moderate or severe OHSS criteria were accepted into the trial, while those who were with clinically diagnosed mild OHSS (only with the abdominal distension, nausea and ovary enlarged, without the above two performance) were excluded in case of interference. After exclusion and screening, the 1780 women were divided into two groups, one group was ET group (n=1195) and the other group was FET group (n=585) on the basis of fresh transplantation or frozen-thawed embryo transfer. According to the criteria we divided those women in the trial into four subgroups: 1) Group A (OHSS-ET, n=126): women who suffered moderate or severe OHSS in their early pregnancy after fresh ET; 2) Group B (non OHSS-ET, n=1069): women without obvious OHSS in their cycle of fresh ET; 3) Group C (OHSS-FET, n=98): women who followed the adoption of the freeze-all strategy in case of the high risk of OHSS while ultimately developed into moderate or severe OHSS after oocyte retrieval; 4) Group D (non OHSS-FET, n=487): women without OHSS after oocyte retrieval and accepting elective FET due to various reasons. Until the study ends, 83 mother and their spontaneously conceived singletons who fulfill the criteria were recruited in the Group E (SC, n=83).

The study investigates the independent effects of ovarian hyperstimulation syndrome on the offspring’s health and development especially the BP level and metabolic function.

Parents gave written informed consent and the study design was approved by the Ethics Committee of the Women’s Hospital, School of Medicine, Zhejiang University.

### Follow-Up Examination

Between 2006 and 2017, we contacted the women whose children were at 3 to 6 years by telephone and invited them to the Reproductive Follow-up clinic of the Women’s Hospital, School of Medicine, Zhejiang University to participate the follow-up assessments. Follow-up work were carryied out on every working day. The trained assessors were blinded to the mode of conception. Each child was requested to come with an empty stomach for the peripheral blood examination in the morning and avoid acute exacerbations of childhood illness including respiratory or intestinal diseases. After a brief introduction on the test to be performed (blood examination, cognitive, cardiovascular and anthropometric assessment), BP and heart rate were measured once. The BP measurement on the non-dominant arm, while the child was seated, was taken with an appropriate cuff size. Then the child would do the peripheral blood examination including the blood routine, blood biochemistry (covering fasting blood glucose, blood fat, hepatorenal function), thyroid function, hepatitis B-virus and trace element examination. It is necessary for the child to have breakfast after finishing the blood examination. Next, BP was measured for the second time. Finally, anthropometric data were collected. In total, BP and heart rate were measured twice. The two readings were averaged to obtain the BP data in mmHg and heart rate in beats/min. In the present paper, cardiovascular, anthropometric outcomes and a part of metabolic function index are reported.

### Statistical Analysis

Group differences in background variables and outcome measures were investigated with Student t-test, Mann-Whitney U tests, Pearson’s Chi-square test when appropriate. In case significant differences were found between two groups, Student’s t-tests and Mann-Whitney U-tests were used to specify the pair-wise differences. At the same time, it was a coincidence between the children’s mother and father in their age in our analysis. Thus, in this study, we did not put father’s age into the multivariable regression analysis.

We performed multivariable linear regression analyses to explore potential differences in BP between the OHSS and non OHSS groups while correcting for possible confounders. In line with other studies in the field (7, 20, 21), the mean differences were adjusted to control for certain confounders according to different models with the linear regression analysis. Models separately were performed for current risk indicators (Weight, gender, age, BMI, pulse and TSH), for early life factors (preterm birth, birth weight, cesarean section), for parental characteristics (maternal age, maternal pre-pregnancy BMI, pregnancy-induced hypertension, gestational diabetes, PCOS), for IVF characteristics (gonadotropin dosage, type of IVF) and finally for all current, early life, parental and IVF variables. Results were expressed as unstandardized regression coefficients (B) with their 95% confidence intervals (95% CI). The analyses were performed using the IBM Statistical Package for the Social Sciences version 23. Probability values of 0.05 were considered statistically significant.

## Results

### Demographics/Maternal Background Data and Subfertility, ART Characteristics

It was shown in **Table 1** of the data of Parental, subfertility and ART characteristics. Most basal characteristics were similar among five or four groups, but several difference were found, like the maternal pregnancy age, the basal FSH or LH levels, estradiol or progesterone level on hCG day, the incidence of PCOS, dosage of GN. Maternal pregnancy age seemed older in the non OHSS-ET group than other four groups. When referring to maternal hormone level, non OHSS-ET group had higher basal FSH and lower basal LH level, lower estradiol level on hCG day. The dosage of gonadotropin was lower in OHSS-ET group as compared with other three groups.

**Table 1:** Demographic data on mothers, subfertility and ART characteristics.

Characteristic	Group A (OHSS-ET)(N = 126)	Group B (non OHSS-ET)(N = 1069)	Group C (OHSS-FET)(N = 98)	Group D(non OHSS-FET)(N = 487)	Group E (SC group)	P values
					(N = 83)	
Maternal characteristics						
Pregnancy age (yr)	29.5 (3.8)	31.0 (3.7)	28.9 (4.0)	30.1 (4.0)	28.07 (3.3)	**<0.001**
BMI before pregnancy^a^ (kg/m^2^)	21.7 (1.0)	21.6 (1.1)	21.5 (0.8)	21.4 (1.3)	21.6 (1.2)	0.178
Basal sexual hormone levels^b^						
FSH (IU/L)	6.2 (1.4)	6.9 (2.2)	5.8 (1.3)	6.4 (2.4)	NA	**<0.001**
LH (IU/L)	5.4 (3.1)	4.9 (2.6)	6.6 (3.3)	4.7 (2.6)	NA	**0.010**
Estradiol (pmol/L)	123.1 (62.1)	177.9 (67.8)	199.2 (69.0)	127.4 (98.0)	NA	0.862
Hormone level on hCG administration day						
Estradiol (pmol/L)	17810 (6825)	11196 (6104)	29129 (8900)	13787 (6556)	NA	**<0.001**
Progesterone (nmol/L)	2.9 (1.2)	2.6 (2.2)	4.3 (2.2)	2.5 (1.3)	NA	**<0.001**
Fertility parameters						
Infertility duration (yr)	3.0 (1.0-9.0)	4.0 (0.5-14.0)	3.8 (1.0-10.0)	4.1 (1.0-15.0)	NA	0.556
PCOS^c^, n (%)	10 (7.9)	47 (4.4)	13 (13.3)shi	31 (6.4)	NA	**0.001**
ART characteristics						
Gn dosage^d^ (IU)	1830 (640)	2273 (897)	1916 (678)	2160 (919)	NA	**<0.001**
Spermatozoa origin (TESA/PESA)^e^, n (%)	9 (7.1)	66 (6.2)	6 (6.9)	22 (4.6)	NA	0.383
ICSI, n (%)	32 (25.4)	281 (26.3)	24 (24.5)	118 (24.2)	NA	0.847

P values were calculated by one-way ANOVA test, Chi-square test.

Statistically significant results (P value<0.05) are displayed in bold numbers. Data represent mean (standard deviation), percentages, or median (range).

a Body mass index was defined as weight divided by height squared.

b The hormone level between the Day1 to Day 3 of the menstrual cycle.

c Polycystic ovary syndrome, diagnosed by the Rotterdam criteria.

d The dose of gonadotrophin during the controlled ovarian hyperstimulation.

e Testicular Sperm Aspiration/percutaneous epididymal sperm aspiration. NA, Not Available.

### Perinatal Characteristics and Blood Pressure, Anthropometrics, Metabolic Function of Offspring

**Tables 2**, **3** showed an overview of all outcome measures for the five groups. As to the obstetric complications, there was no significant difference among groups in the incidence of complications such as gestational hypertension, gestational diabetes. The cesarean section was more often performed in the ART groups than the SC group. Children born to non OHSS-FET had a higher weight, BMI than other groups. SBP, DBP and heart rate were differ among five groups. Metabolic index including fasting blood glucose, serum lipid, and thyroid function seemed no significantly difference.

**Table 2:** Demographic data on children and perinatal characteristics.

Characteristic	Group A (OHSS-ET)(N = 126)	Group B (non OHSS-ET)(N = 1069)	Group C (OHSS-FET)(N = 98)	Group D (non OHSS-FET)(N = 487)	Group E (SC group)	P values
					(N = 83)	
Gestational characteristics						
Gestational diabetes, n (%)	9 (7.1)	95 (8.9)	9 (9.2)	48 (9.9)	7 (8.4)	0.907
Pregnancy-induced hypertension, n (%)	4 (3.2)	73 (6.8)	10 (10.2)	33 (6.8)	3 (3.6)	0.123
Preeclampsia, n (%)	3 (2.4)	70 (6.5)	9 (9.2)	32 (6.6)	1 (1.2)	0.072
Perinatal characteristics						
Preterm birth (<37week), n (%)	11 (8.7)	120 (11.2)	9 (9.2)	52 (10.7)	6 (7.3)	0.728
Birth weight (g)	3306 (525)	3252 (543)	3388 (424)	3530 (373)	3305 (653)	0.915
Cesarean section, n (%)	93 (73.8)	923 (86.3)	81 (82.7)	444 (91.2)	44 (53.0)	**<0.001**
Children characteristics						
Age (yr)	4.1 (0.3)	4.1 (0.4)	4.1 (0.4)	4.1 (0.2)	4.3 (0.6)	0.357
Male gender, n (%)	70 (55.6)	549 (51.4)	44 (44.9)	251 (51.5)	50 (60.2)	0.283
Standing Height (cm)	109.0 (99.0-116.0)	109.0 (94.3-130.0)	108.4 (102.0-116.2)	110.8 (102.0-120.0)	109.0 (97.5-124.5)	0.241
Weight (kg)	17.6 (14.4-28.0)	18.0 (12.7-31.8)	18.4 (15.3-22.0)	19.6 (15.2-28.5)	17.8 (14.3-29.0)	**0.040**
BMI^a^ (kg/m^2^)	15.0 (13.3-20.8)	15.1 (12.4-23.2)	15.6 (13.9-19.4)	16.0 (13.1-22.1)	14.9 (12.6-19.9)	**0.023**
BMI>18kg/m^2^, n(%)	11 (8.7)	65 (6.1)	5 (5.1)	45 (9.2)	4 (4.8)	0.142

P values were calculated by one-way ANOVA test, Chi-square test. Statistically significant results (P value<0.05) are displayed in bold numbers. Data represent mean (standard deviation), percentages, or median (range).

a Body mass index was defined as weight divided by height squared.

**Table 3:** Blood pressure (in mmHg) and metabolic function for 3 to 6-year-old singletons born after IVF/ICSI with OHSS-ET (Group A), non OHSS-ET (Group B), OHSS-FET (Group C) or non OHSS-FET (Group D) and SC (Group E).

Characteristic	Group A (OHSS-ET)(N = 126)	Group B (non OHSS-ET)(N = 1069)	Group C (OHSS-FET)(N = 98)	Group D (non OHSS-FET)(N = 487)	Group E (SC group)	P values
					(N = 83)	
Blood pressure, pulse pressure, and heart rate						
SBP (mm Hg)	101.93 (8.17)	99.49 (8.91)	99.38 (8.17)	99.56 (8.50)	99.84 (8.9)	**0.048**
DBP (mm Hg)	58.75 (8.48)	56.55 (8.02)	55.72 (7.94)	56.14 (7.77)	55.27 (8.8)	**0.008**
SBP>110mmHg, n(%)	20 (15.9)	117 (10.9)	9 (9.2)	45 (9.2)	6 (7.2)	0.199
DBP>70mmHg, n(%)	7 (5.6)	16 (1.5)	6 (6.1)	4 (0.8)	3 (3.6)	**<0.001**
Pulse pressure (mm Hg)	42.10 (8.60)	43.12 (9.33)	42.06 (8.15)	42.74 (9.50)	44.58 (8.88)	0.481
Heart rate (beats/min)	95.23 (69-124)	97.17 (70-126)	92.29 (69-110)	99.66 (75-122)	93.98 (70-122)	**0.004**
Fasting blood-glucose (mmol/L)	4.95 (0.52)	4.87 (0.39)	4.85 (0.41)	4.85 (0.43)	4.84 (0.4)	0.428
Serum lipid						
Triglyceride (mmol/L)	0.65 (0.50-1.62)	0.69 (0.35-3.34)	0.61 (0.36-0.84)	0.70 (0.36-1.70)	0.66 (0.36-1.53)	0.065
Total cholesterol (mmol/L)	4.49 (1.12)	4.29 (0.76)	4.29 (0.62)	4.25 (0.72)	4.34 (0.73)	0.847
LDL (mmol/L)	2.40 (0.94)	2.23 (0.60)	2.05 (0.46)	2.16 (0.58)	2.30 (0.61)	0.826
HDL (mmol/L)	1.42 (0.32)	1.44 (0.30)	1.51 (0.26)	1.41 (0.32)	1.40 (0.28)	**0.049**
Uric acid (μmol/L)	265.55 (58.09)	251.58 (51.58)	261.00 (54.17)	266.72 (49.47)	262.90 (56.72)	0.532
TSH (mIU/L)	1.98 (1.23-4.84)	2.23 (0.32-8.41)	2.32 (1.00-5.73)	2.17 (0.72-4.91)	2.21 (0.86-7.71)	0.063

P values were calculated by one-way ANOVA test, Chi-square test. Statistically significant results are displayed in bold numbers. Data represent mean (standard deviation), percentages, or median (range).

SBP, systolic blood pressure; DBP, diastolic blood pressure; Pulse pressure: SBP minus DBP; LDL, low density lipoprotein; HDL, high density lipoprotein; TSH, thyrotropin hormone.

A single factor analysis was performed for the SC with ART groups. The BP in the SC group was lower than the OHSS-ET group while similar with other 3 groups, which was showed in the **Table 4**.

**Table 4:** Comparison the BP of SC (group E) and ART group.

Covariate	Reference	SBP mean difference (95% CI) unadjusted	P value	DBP mean difference (95% CI) unadjusted	P value
SC (E, n = 83)	OHSS-ET (A, n = 126)	-2.097 (-4.930 to -0.090)	**0.042**	-3.481 (-5.700 to -1.260)	**0.002**
	non OHSS-ET (B, n = 1069)	-0.064 (-2.010 to 1.890)	0.949	-1.284 (-3.080 to 0.510)	0.160
	OHSS-FET (C, n = 98)	0.044 (-2.510 to 2.600)	0.973	0.701 (-2.810 to 1.890)	0.701
	non OHSS-FET (D, n = 487)	-0.137 (-2.170 to 1.890)	0.895	-0.873 (-2.740 to 0.990)	0.360

P value were calculated by one-way ANOVA test.

Statistically significant results are displayed in bold numbers.

### Multiple Regression Analysis on the Blood Pressure of 3 to 6-Year-Old Singletons

Subsequently, we performed multiple linear regression analysis of SBP and DBP among the ART groups in mmHg showed in **Table 5**. Children born following OHSS-ET had higher SBP and DBP in mmHg than children born following non OHSS-ET and OHSS-FET, also after correction for various sets of variables. While in the comparison of non OHSS-FET with non OHSS-ET, the blood pressure showed no obvious and unstable differences after adjusting those concomitant variables.

**Table 5:** Multiple linear regression analysis of the effect of OHSS on blood pressure.

Model	Covariate	Reference	SBP mean difference (95% CI) unadjusted	P value	DBP mean difference (95% CI) unadjusted	P value
Unadjusted	OHSS-ET (A)	non OHSS-ET (B)	2.443 (0.830 to 4.050)	**0.003**	2.197 (0.720 to 3.680)	**0.004**
		OHSS-FET (C)	2.551 (0.250 to 4.850)	**0.021**	3.022 (0.900 to 5.140)	**0005**
	non OHSS-FET (D)	non OHSS-ET (B)	0.073 (-0.860 to 1.010)	0.878	-0.412 (-1.263 to 0.440)	0.348
Current risk indicators	OHSS-ET (A)	non OHSS-ET (B)	3.306 (0.574 to 2.249)	**0.001**	3.260 (0.507 to 2.038)	**0.001**
		OHSS-FET (C)	2.142 (0.185 to 4.450)	**0.033**	2.889 (1.006 to 5.330)	**0.004**
	non OHSS-FET (D)	non OHSS-ET (B)	-0.369 (-1.268 to 0.531)	0.421	-0.838 (-1.651 to -0.025)	0.043
Early life factors	OHSS-ET (A)	non OHSS-ET (B)	3.046 (0.463 to 2.139)	**0.002**	3.077 (0.435 to 1.966)	**0.002**
		OHSS-FET (C)	2.339 (0.407 to 4.776)	**0.020**	2.872 (1.018 to 5.470)	**0.004**
	non OHSS-FET (D)	non OHSS-ET (B)	0.070 (-0.875 to 1.015)	0.885	-0.420 (-1.274 to 0.434)	0.335
Parental characteristic	OHSS-ET (A)	non OHSS-ET (B)	3.178 (0.521 to 2.201)	**0.002**	3.251 (0.506 to 2.045)	**0.001**
		OHSS-FET (C)	2.210 (0.370 to 6.604)	**0.029**	1.787 (-0.307 to 6.103)	0.076
	non OHSS-FET (D)	non OHSS-ET (B)	-0.202 (-1.686 to 1.282)	0.789	-0.429 (-1.761 to 0.904)	0.528
IVF characteristic	OHSS-ET (A)	non OHSS-ET (B)	2.910 (0.415 to 2.132)	**0.004**	3.028 (0.429 to 2.010)	**0.003**
		OHSS-FET (C)	2.420 (0.726 to 7.198)	**0.017**	1.293 (-1.137 to 5.442)	0.198
	non OHSS-FET (D)	non OHSS-ET (B)	-0.317 (-1.835 to 1,201)	0.682	-0.649 (-2.020 to 0.722)	0.353
All above	OHSS-ET (A)	non OHSS-ET (B)	3.193 (0.549 to 2.301)	**0.001**	3.440 (0.611 to 2.233)	**0.001**
		OHSS-FET (C)	2.147 (0.290 to 7.271)	**0.034**	0.747 (-2.269 to 5.012)	0.457
	non OHSS-FET (D)	non OHSS-ET (B)	-1.187 (-2.674 to 0.299)	0.117	-1.213 (-2.572 to 0.145)	0.080

Data is represented by the mean difference (unadjusted 95% confidence interval). P values were calculated by multiple regression analysis. Statistically significant results (P value<0.05) are displayed in bold numbers.

SBP, systolic blood pressure; DBP, diastolic blood pressure.

Current risk indicators: adjust standing height, weight, gender, age, BMI, pulse, and TSH of the child for the BP analysis.

Early life factors: adjust preterm birth, birthweight, and cesarean section.

Parental characteristics: adjust maternal age, maternal BMI before pregnancy, pregnancy-induced hypertension, gestational diabetes, PCOS, infertility duration and type of infertility for the BP analysis.

IVF characteristic: adjust for the dosage of gonadotrophin, ICSI, Spermatozoa origin.

## Comment

It is a large sample and prospective study to investigate the effect of OHSS on offspring’s health. Evidence is accumulating in animals and humans that ART alters the cardiovascular phenotype. For example, there is evidence in normal mice that ART causes premature vascular aging and arterial hypertension that is related to an epigenetic mechanism and associated with a shortened life span (22). In line with these findings, studies demonstrate that ART has shown to cause morphological alteration of the vascular in the systemic circulation (9) and increase arterial BP (7, 20) in apparently healthy ART children. Although variety studies demonstrating ART-induced alterations of the offspring phenotype, it is lack of the researches on a large sample or adjusting multiple relative factors like ours. This study showed that children born from OHSS mothers might have higher BP than SC children or non-OHSS children. After adjusting other risk factors, OHSS-ET children also showed significantly higher blood pressure when compared with non OHSS-ET children. Meanwhile, to our knowledge, it’s the first follow-up study that set the OHSS-FET and non OHSS-FET as the control group to confirm the benefits of freeze-all strategy for those at high risk OHSS after oocyte retrieval and their offspring. The BP displayed no obvious differences when compared SC with non-OHSS ET/FET, non OHSS-FET with non OHSS-ET. Through setting multiple control groups and preforming multiple regression analysis, our study indicated that OHSS in early pregnancy might play an impendent key role on offspring’s BP even cardiovascular function. As we known, an OHSS pregnancy compared with a spontaneously conceived pregnancy showed high estrogen and progesterone in early pregnancy (16). To some extent, it indicates that higher estrogen and progesterone probably influence the offspring’s cardiovascular. In the present study, we found that 4-year-old IVF children born after ovarian stimulation have higher blood pressure (23). OHSS children showed an alteration of cardiovascular functions, which may be related to the mother’s super-physiological dose of estrogen and progesterone (17). To date, the association between prenatal exposure of high estradiol/progesterone and cardiovascular changes of offspring is still unclear. Also, the angiotensin II was significantly increased in the ascites of OHSS pregnancy (24). Angiotensin II is been considered as a consensus cardiovascular factor. The mechanism under those researches needs further data support and experimental research.

From a clinical point of view, it may seem like a tiny gap that OHSS children have 2 to 4-mm Hg higher systolic and diastolic blood pressure than non OHSS children. And researches shows the prevalence of hypertension in children is low: for example, a study conducted in Switzerland suggested a prevalence of only 2 % (25). However, a slight increase in blood pressure may significantly raise the risk of future cardiovascular disease. Hypertension is a major risk factor for coronary heart disease, peripheral artery occlusive disease, stroke, and even chronic kidney disease (26–29). For instance, lowering mean systolic blood pressure in adults by 2 mm Hg corresponds to an 8% reduction in the risk of stroke. Furthermore, it cannot be excluded that increased blood pressure after IVF may be amplified throughout life because blood pressure is known to track from childhood into adult life (30, 31).

Existing research shows that use of hCG to trigger ovulation or luteal support was significantly associated with early-onset OHSS (32–34). Elevate endogenous hCG in early pregnancy is associated with late-onset OHSS. Selective embryo freezing is a routine method for preventing late-onset OHSS by avoiding continued exposure to hCG during the luteal phase, avoiding cycle cancellation, and guaranteeing cumulative pregnancy rates. Current clinical pregnancy rates for FET cycles are comparable to fresh embryo transfer cycles (35). Although selective embryo freezing cannot completely avoid the occurrence of early-onset OHSS, it can more effectively reduce the incidence of early-onset OHSS and avoid the occurrence of late-onset OHSS compared with other methods. Therefore, the establishment of the FET group in our study has certain practical significance.

The purpose of this study was primarily to focus on the health of ART offspring. Therefore, the follow-up nodes set up cover almost the entire period of children, including birth, infancy, preschool, school, and adolescence. Follow-up studies have been ongoing for more than 10 years and are continuing. This sub-project study focuses on the cardiovascular and metabolic changes of the offspring. The starting point of the first study is in the preschool age (3 to 6 years old). And a series of follow-up studies will be carried out in the future, aiming to discover the dynamic changes of the relevant indicators throughout childhood and even adulthood through very early follow-up examinations.

When interpreting our study, the limitations of our study also need to be considered. In our study, BP was measured in office twice on one day and the mean of the two measurements was used for analysis. This may deviate the final blood pressure value from the actual value and miss differences between groups. Similarly, we found other published studies in the area also have the difficulty to measure the BP multiple times. Studies evaluating the effects of reproductive technologies most opted to measure BP on a single day (7, 8, 20). Using gold standard assessment (24h-ambulatory blood pressure measurements, ABPM) of arterial blood pressure, evidence for increased blood pressure in young adult ART participants have been presented at the ESC meeting (36). Such measurements, in addition to settling the issue of ART-induced hypertension in humans, also would provide important information on additional independent predictors of cardiovascular risk (i.e. night-time dipping, blood pressure variability). In future research, we will consider using ABPM in the hope of getting more accurate cardiovascular data.

There is abundant evidence that essential hypertension is a highly heritable condition. Although at the beginning of the research, we had excluding the couple who had the history of hypertension, diabetes, and family history of those diseases, we did not collect the basic blood pressure indicators of both husband and wife. It, therefore, appears possible that BP in the parents of Group A is higher than in the parents of the other groups and that they transmit this trait to the offspring. Additionally, although the pregnancy-induced hypertension is as a well-established cardiovascular risk factor in naturally conceived humans, the effect of the ART combined with pregnancy-induced hypertension on the blood pressure of the offspring was not studied separately in this research. We put the pregnancy-induced hypertension as a factor into the multiple regression analysis that showed pregnancy-induced hypertension also affect offspring blood pressure (data was not labeled).

In addition, there were some differences in the baseline data among these groups, which may seem to affect the outcomes. First women in the SC group seemed much younger than the ART group. Since the SC group is a randomly selected voluntary participant who meets the criteria, the age of infertile patients is generally larger than that of non-infertile patients in the clinical status. In the OHSS group, the basal LH value of the mother was higher than that in the control group, and the basal FSH value was also increased in the non OHSS group. It was consistent with clinical that the ovarian function of the OHSS patients was superior to that of the non-OHSS women. It can also explain the result that mothers in the OHSS group were younger than those in the control group, as the ovarian function is known to decrease linearly with age. At the same time, the higher total dose of Gn during ovulation induction in the OHSS group can be fully explained from a clinical point of view. Out of clinician’s experience, younger infertile women of better ovarian function often take the ovulation induction programs in low doses to avoid OHSS although eventually developed into OHSS. In our study, we took multivariable regression analyses to assess the outcome to control these confounders. In another way, it also reflects the reliability and authenticity of the data in this study. In addition, we also did a propensity matching analysis for Group A (OHSS-ET) and Group B (non OHSS-ET). Although the sample size after matching (maternal age, basal FSH and trigger day estradiol levels) was only 76 cases in each group, the final univariate analysis showed that Children had higher BP in the OHSS-ET group than in the non OHSS-ET (SBP: 101.79 ± 8.02 vs. 98.63 ± 8.34, P=0.019; DBP: 59.87 ± 8.18 vs. 56.18 ± 7.47, P=0.004). There were no significant differences in other metabolic indicators between the two groups.

The study also included IVF and ICSI children. As it is unknown whether IVF and ICSI share the same cardiometabolic risk for offspring (20), we performed a linear regression analysis. Indeed, several studies suggested cardiometabolic alterations in IVF/ICSI offspring (5, 6, 8, 9).

It can be found that the cesarean section rate in our study was extremely high. The main reason was that the offspring conceived through IVF/ICSI was viewed as “precious child”. Most parents view cesarean section as a safer way to deliver. There is also a focus on having a “perfect baby” under the “one child policy” in the past (37).

In conclusion, the results of the present study suggest that OHSS in the early pregnancy is associated with higher blood pressure in mmHg including systolic blood pressure and diastolic blood pressure in 3 to 6-year-old offspring. While electing frozen-thawed embryo transfer for high risk of OHSS population may reduce the risk of that high BP trend. In addition, we found no significant evidence for an adverse effect of OHSS on anthropometrics and metabolic function. Future research is needed to confirm the role of OHSS or early environment for oocyte and embryo in a poorer cardiometabolic outcome and investigate the underlying mechanisms. Our findings emphasize the importance of the accuracy of the IVF clinic strategy and preventing the OHSS after fresh embryo transfer, calling for the cardiovascular monitoring of the growing number of children conceived with IVF worldwide.

## Data Availability Statement

The original contributions presented in the study are included in the article. Further inquiries can be directed to the corresponding author.

## Ethics Statement

The studies involving human participants were reviewed and approved by the Ethics Committee of the Women's Hospital, School of Medicine, Zhejiang University. The participants provided their written informed consent to participate in this study.

## Author Contributions

All authors fulfil the criteria for authorship; YZ initiated the study, YF, MT and HY collected the data. YF interpreted and analysed the data, who finally drafted the report. All authors commented on the drafts, and have seen and approved the final version.

## Funding

The study was supported by the Ministry of Health of PRC Science Foundation (NO. WKJ-ZJ-1522), the Natural Science Foundation of Zhejiang Province (No. LZ15H040001), the National science & Technology Pillar Program during the 13th Five-year Plan Period (NO. 2016YFC1000302), and the Zhejiang Provincial & Ministry of Health Research Fund For Medical Sciences WKJ-ZJ-1722, and the Program for Key Subjects of Zhejiang Province in Medicine & Hygiene. The sponsors of the study had no role in study design, data collection, data analysis, data interpretation, or writing of the report.

## Conflict of Interest

The authors declare that the research was conducted in the absence of any commercial or financial relationships that could be construed as a potential conflict of interest.

## Publisher’s Note

All claims expressed in this article are solely those of the authors and do not necessarily represent those of their affiliated organizations, or those of the publisher, the editors and the reviewers. Any product that may be evaluated in this article, or claim that may be made by its manufacturer, is not guaranteed or endorsed by the publisher.
